# Anti-hypercholesterolemic Effect of Berbamine Isolated from *Rhizoma Coptidis* in Hypercholesterolemic Zebrafish Induced by High-Cholesterol Diet

**Published:** 2018

**Authors:** Bing Han, Shuming Kou, Kai He, Yulong Han, Yue Wang, Tao Huang, Xia Zhou, Yubo Xiao, Xuegang Li, Xiaoli Ye

**Affiliations:** a *School of Life Sciences, Southwest University, Chongqing, 400715, China. *; b *School of Pharmaceutical Sciences, Southwest University, Chongqing, 400715, China. *; c *Chongqing productivity promotion center for the modernization of Chinese traditonal medicine, Chongqing, 400716, China*.

**Keywords:** Berbamine, *Rhizoma Coptidis*, Zebrafish, Hypercholesterolemia, Vascular lipid accumulation

## Abstract

The anti-hypercholesterolemic effect of berbamine (BBM) isolated from *Rhizoma Coptidis* (RC) was investigated in hypercholesterolemic zebrafish model induced by high-cholesterol (HC) diet. Zebrafish embryo assay revealed no significant difference in morphology and cell death with the treatment of BBM less than 20 μg/mL. In zebrafish larvae, the fluorescently labeled cholesterol in caudal artery was reduced dose-dependently after BBM treatment. For adult zebrafish, administration of 0.2% BBM exhibited a significant decrease in plasma total cholesterol (TC), triglyceride (TG) and low-density lipoprotein cholesterol (LDL-c) levels by 37%, 38% and 28%, respectively, along with a fall in lipid content in liver. Further investigation suggested that the mRNA expression of 3-hydroxy-3-methylglutaryl-coenzyme A reductase (HMGCR) and microsomal triglyceride transfer protein (MTP) in liver were down-regulated and the transcription levels of liver gene low-density lipoprotein receptor (LDLR) and cytochrome P450 polypeptide 1a of subfamily A of family 7 (CYP7A1a) were significantly up-regulated with BBM treatment. Histological study showed that BBM can alleviate hepatic steatosis induced by HC diet. These data suggested that BBM has anti-hypercholesterolemic and hepatoprotective effects. The mechanism probably related to the up-regulation of cholesterol transport and bile acid synthesis as well as inhibition of cholesterol synthesis and lipoprotein assembly or secretion.

## Introduction

Cardiovascular disease is the main cause of morbidity and mortality worldwide. Besides many environmental and genetic risk factors that have been demonstrated by epidemiologic studies, dyslipidemia, which is associated with abnormal lipid levels mainly increased serum cholesterol, is a major risk factor for the development of cardiovascular disease (1-3). It is acknowledged that controlling serum cholesterol level can effectively reduce hypercholesterolemia and attenuate the progression of atherosclerosis (4, 5).

*Rhizoma Coptidis* (RC) is the root of *Coptis chinensis *Franch, family *Ranunculaceae*. The pharmacological function of RC was first recorded in Shen Nong Ben Cao Jing for its heat-clearing and detoxifying function. The crude extract of RC consisted of main alkaloids and minor alkaloids. The total content of main alkaloids was up to 90% of crude extract, and that of minor alkaloids was about 10%. In fact, RC consisted of main alkaloids including berberine, coptisine, palmatine, epiberberine and jatrorrhizine, and minor alkaloids such as berbamine (BBM), 8-O-coptisine, 8-O-berberine, groenlandicine, and berberrubine (6). In our previous studies, we mixed five main alkaloids as a combination (marked as COM) in a proportion of which were in RC (7). It demonstrated that COM showed a higher cholesterol-lowering efficacy than single main alkaloids. However, the cholesterol-lowering effect of COM was still lower than the crude extract of RC (not published). As there were still minor alkaloids in crude extract of RC. Thus, we speculated these minor alkaloids might possess or contribute in the lipid-lowering efficacy. 

BBM is one of the minor ingredients in RC (8, 9). Studies noted that BBM exhibited a variety of biological activities such as anti-inflammatory, anti-nociceptive, anti-pyretic, and anti-tumor activities (10-13). When it comes to cardiovascular pharmacology, previous studies suggested that BBM exhibited anti-arrhythmia, anti-myocardial ischemia, anti-hypertension, and anti-thrombosis effects (14). However, few researches have been carried out to investigate the anti-hypercholesterolemic effect of BBM.

As a vertebrate, zebrafish (*Danio rerio*) has a high (80-85%) genetic homology to human (15). Zebrafish produces a large number of offspring at low cost which makes large-scale drug screens possible. Additionally, the optical transparency of the larvae together with the fluorescent probes enables the direct visualization of complex phenomena at the level of the entire organism (16-18). Consequently, zebrafish is increasingly being used as a model of human diseases as well as for high-throughput screening (19-21). Stoletov *et al*. further showed that zebrafish fed with high-cholesterol (HC) diet developed hypercholesterolemia and accumulated vascular lipids (17). Together with the fact that teleost fish shares a high degree of conservation of lipid metabolism with mammals (22, 23), it is likely that compounds evaluated in a zebrafish model will act through comparable mechanisms in mammals.

In this study, the hypocholesterolemic activity of BBM on zebrafish fed with HC diet was evaluated and the underlying mechanism of lipid-lowering effect was also investigated by detecting the mRNA expression of genes involved in cholesterol metabolism.

## Experimental

*Plant and preparation of BBM*

RC was obtained from Good Agricultural Practices Demonstration Base in Shizhu city (Chongqing, China) and authenticated by Prof. Lvjiang Yuan at Southwest University. The voucher specimen (No. 20130315) was deposited at the herbarium of the Study and Development Center of Huanglian, College of Pharmaceutical Sciences, Southwest University. The dried RC powder (5 kg) was extracted three times with 70% ethanol at room temperature and then filtered. The filtrate was evaporated at 60 C in a rotary evaporator under reduced pressure to obtain 1.25 kg of crude extract (A). Then the extract was dissolved in 2% HCl solution and ultrasonically extracted for 2 h. After centrifuged at 5000 rpm for 10 min, the supernatant was adjusted with ammonium hydroxide to pH 10.0, and then extracted with chloroform and n-butanol. Our previous results showed that five main alkaloids were distributed in n-butanol. And BBM was mainly in chloroform fraction. Therefore, the chloroform fraction (B) was evaporated and loaded onto 200 g of silica gel (200-300 mesh), and gradiently eluted with EtOAc-petroleum ether-methanol (1:5:0-0:0:1, V/V/V) and petroleum ether-chloroform-methanol (5:1:0-0:0:1, V/V/V) respectively, and further purified with Sephadex LH-20 to obtain five compound fractions, including BBM, 8-O-coptisine, 8-O-berberine, groenlandicine and berberrubine. The fraction of BBM was recrystallized to obtain pure BBM (C) in methanol solution. 

The purified compounds were identified by comparing their melting points, UV, ^1^H and ^13^C NMR data with the literature. The purity of BBM was detected by high-performance liquid chromatography (HPLC, Shimadzu LC-20A, Japan) equipped with a Hypersil C18-ODS (4.6 mm ×200 mm, 5 μm) under following analyzing conditions: mobile phase, acetonitrile: 0.2% triethylamine = 82:18; flow rate, 1 mL/min; injection volume, 20 μL; wavelength of detection, 281 nm; temperature of column, 40 °C.

*Reagents*

Commercial kits for analysis of total cholesterol (TC), triglyceride (TG), low-density lipoprotein cholesterol (LDL-c) and high-density lipoprotein cholesterol (HDL-c) were purchased from Changchun Huili Biotech Co., Ltd (Changchun, China). Trizol Reagent and fluorescent cholesteryl BODIPY^®^ 542/563 C11 were from Invitrogen Co. (California, USA). SYBR Green Supermix was obtained from Bio-Rad Laboratories (California, USA). Other reagents were purchased from Beijing Dingguo Changsheng Biotechnology Co., Ltd (Beijing, China).

*Origin and maintenance of zebrafish*

Adult zebrafish were kept at 28 °C under a 14-h light/10-h dark cycle, and water conditions were maintained according to the *Zebrafish Book* (24). Embryos were obtained from natural spawning that was induced in the morning by turning on the light. The embryos were collected within 30 min and the normally developed embryos, those had reached the blastula phase were incubated in the embryo medium (0.2 mM Ca (NO_3_)_2_, 0.13 mM MgSO_4_, 19.3 mM NaCl, 0.23 mM KCl, and 1.67 mM HEPES) for further analysis (25). Zebrafish maintenance and experimental procedures were approved by the Animal Care and Use Committee of Southwest University. All surgery was performed under anesthesia and all efforts were made to minimize suffering.

*Food preparation*

The control diet was a standard zebrafish chow (Zebrafish feed: 46.8% crude protein, 5.2% crude fat, 10.0% crude fiber, 10.5% crude ash, vitamin A (11000 IU/kg), vitamin D3 (2300 IU/kg) and vitamin E (270 mg/kg), Taikong Co., Ltd., batch number 20140823). 

The HC diet containing 4% cholesterol was made by soaking control diet in a diethyl ether solution of cholesterol after ether evaporation (17). The BBM diet was prepared by adding 0.05%, 0.1% and 0.2% (w/w) of BBM to HC diet (marked as BBML, BBMM and BBMH, respectively). To measure the lipid content of the standard zebrafish chow, lipids were extracted using the conventional Folch method (26) and it showed that the cholesterol and triglyceride content in the control diet was 0.4% (w/w) and 1.0% (w/w), respectively.

*Waterborne exposure of zebrafish embryos to BBM*

From approximately 3 hours post-fertilization (hpf), embryos were transferred to individual wells of a 24-well plate and maintained in embryo medium. The embryos were treated with 0, 5, 10, 20, 40 or 80 μg/mL BBM and developed up to 5 days post-fertilization (dpf).

*Acridine orange staining of zebrafish embryos*

Acridine orange is a nucleic acid selective metachromatic dye which interacts with DNA and RNA by intercalation or electrostatic attractions. Acridine orange stains cells with disturbed plasma membrane permeability so it preferentially stains necrotic or apoptotic cells. At 3 hpf, the embryos were treated with 0, 5, 10, 20, 40 or 80 μg/mL BBM in a 24-well plate and developed up to 48 hpf. Then the embryos were incubated with acridine orange solution (7 μg/mL) for 30 min in dark at 28 °C as described previously (25). After incubation, the embryos were washed in embryo medium and anaesthetised before visualisation. The images of stained embryos were observed using a fluorescence microscope (Nikon Eclipse Ci, Japan). The fluorescence intensity of individual embryo was quantified using Nikon Elements-DR software.

*Measurement of heartbeat rate*

The heartbeat rate of both atrium and ventricle were measured at 5 dpf to determine the toxicity of compound. Counting and recording of atrial and ventricular contractions were performed for 3 min under a microscope and the results were presented as the average heartbeat rate per min (27).

*Measurement of vascular lipid accumulation in zebrafish larvae*

At 5 dpf, zebrafish larvae were fed with the control, the HC or the HC diet with 5, 10 or 20 μg/mL of BBM twice daily for 10 days at 28 °C under light-shielded conditions, all diets were supplemented with 10 μg/g red fluorescent cholesteryl BODIPY^®^ 542/563 C11. The fish were allowed to consume their diet for 30 min and the water in each dish was replaced with fresh water after each feeding. On the final day of the experiment, 3 larvae from each group were randomly selected and anesthetized. Then the caudal artery of larvae was immediately photographed using a fluorescence microscope (28). The fluorescence intensity of the caudal artery was quantified by Nikon Elements-DR software.

*Measurement of body weight and plasma lipid levels in adult zebrafish*

At 2.5 months post-fertilization (mpf), zebrafish were weighed under anesthesia and randomly divided into NC, HC and BBM groups (n = 20) with similar body weights. Zebrafish in groups were fed with the control, HC, BBML (0.05%), BBMM (0.1%) and BBMH (0.2%) diet, respectively. Each group was fed three times daily (15 mg of food/day/fish) for 4 weeks. During feeding, water inflow to the tanks was paused until the food was eaten up. Body weight and length of zebrafish were recorded at the end of the experiment. Zebrafish length was measured from the head to the end of the body (19). For the analysis of plasma lipid levels, zebrafish were fastted overnight and blood was withdrawn from the dorsal artery of the zebrafish at the indicated times as described previously with minor modifications (29). Plasma was obtained by centrifugation of blood samples at 3500 rpm for 5 min at room temperature. Liver lipids were extracted using the conventional Folch method (26). Then the levels of plasma TC, TG, LDL-c and HDL-c as well as hepatic lipid levels were enzymatically determined using a commercial kit according to the manufacturer’s instruction.

*Zebrafish anesthesia*

To get live images, larval zebrafish was anesthetized by being exposed to 0.02% tricaine prior to microscopic observation (28). For adult zebrafish, ice chips were added to aquarium water and the temperature should be about 4 °C. Zebrafish were anesthetized in chilled water until it no longer responds to external stimuli as described previously (29).

*RNA extraction and quantitative real-time PCR*

Total RNA was extracted from the livers of fish in all groups using Trizol reagent. cDNA was synthesized using random primers and SuperScript III First-Strand Synthesis System. Quantitative real-time PCR, containing target genes and SYBR Green Supermix, was performed on Bio-Rad CFX real-time system (Bio-Rad, USA) in triplicate according to manufacturer’s protocol. The thermal cycling condition comprised an initial step at 95 °C for 3 min followed by 40 cycles of 95 °C for 10 sec, 55 °C for 30 sec and 72 °C for 30 sec. The primers used in quantitative real-time PCR were shown in Table 1. Relative mRNA expression levels were determined by using the expression level of GAPDH as an internal standard.

*Oil red O staining*

For the observation of lipid accumulation, livers were collected from zebrafish by surgical manipulation under a stereoscopic microscope (Leica MZ16F, Germany). Flash-frozen in liquid nitrogen embedded in Tissue-Tek then sliced to a thickness of 10 μm using a microtome (Leica CM1900, Germany) at -20 °C. Air-dried sections were fixed in 10% buffered formalin solution at 4 °C for 2 h rinsed in distilled water and subsequently in 60% isopropanol for a few seconds. The sections were then immersed in a working solution of Oil red O for 15 min and rinsed with distilled water. Sections were also counterstained using Mayer’s hematoxylin to visualize the nuclei as described previously (30). Morphological changes were compared using a microscope.

*Statistical analysis*

All data were expressed as means ± SE. Statistical analysis was performed using SPSS 18.0. Differences between the means were evaluated via one-way ANOVA and post hoc analysis of group differences was performed by least significant difference (LSD) test. Differences between groups were considered significant at *p *<0.05.

## Results

*Structure of BBM*

BBM: white amorphous powder. ^1^HNMR (500 MHz, CDCl_3_): δ 3.90 (m, JH, H-1), 2.25 (s, 3H, 2-Me), 6.26 (s,1H, H-5), 6.42 (d, 1H, J = 2 Hz, H-10), 6.83 (d, 1H, J = 8 Hz, H-13), 6.73 (dd, 1H, J = 8, 2Hz, H-14), 3.95 (m, 1H, H-1’), 2.60 (s, 3H, 2’-Me), 6.51 (s, 1H, H-5’), 5.98 (br, 1H, H-8’), 6.42 (dd, 1H, J = 8, 2Hz, H-10’), 6.62 (dd, 1H, J = 8, 2Hz, H-11’), 7.10 (dd, 1H, J = 8, 2Hz, H-13’), 7.30 (dd, 1H, J = 8, 2 Hz, H-14’), 3.75 (s, 3H, 6-OMe), 3.18 (s, 3H, 7-OMe), 3.58 (s, 3H, 6’-OMe); ^13^CNMR (CD_3_Cl_3_, 12Hz): δ 62.7 (C-1), 43.0 92-Me), 46.0 (c-3), 25.5 (C-4), 128.9 (C-4a), 105.8 (C-5), 152.c (C-6), 137.3 (C-7), 147.0 (C-8), 121.8 (C-8a), 38.1 (C-α), 134.0 (C-9), 115.9 (C-10), 144.6 (C-11), 148.2 (C-12), 115.7 (C-13), 123.9 (C-14), 64.0 (C-1’), 43.1 (2’-Me), 46.0 (C-3’), 25.5 (C-4”), 127.0 (C-4’a), 115.5 (C-5’), 150.3 (C-6’), 143.8 (C-7’), 120.3 (C-8’), 38.6 (C-α’), 132.7 (C-9’), 130.6 (C-10’), 12.1 (C-11’), 154.5 (C-12’), 121.7 (C-13’), 130.6 (C-14’), 56.2 (6-OMe), 60.9 (7-OMe), 55.9 (6’-OMe). 

**Figure 1:**
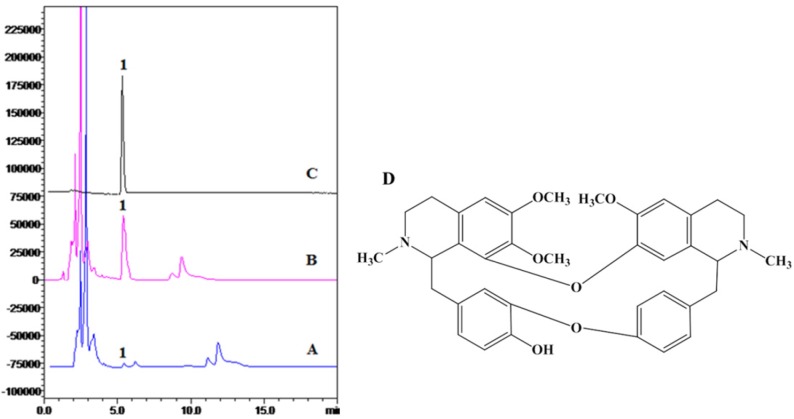
HPLC analysis and structure of BBM. A: ethanol crude extract; B: chloroform extract; C: crystal compound of BBM fraction; D: structure of BBM. HPLC detection condition: Hypersil C18-ODS column (4.6 mm  200 mm, 5 μm); mobile phase, acetonitrile: 0.2% triethylamine = 82:18; flow rate, 1 mL/min; injection volume, 20 μL; wavelength of detection, 281 nm; temperature of column, 40  C

The ^1^HNMR and ^13^CNMR spectra were equal to previous reports (31, 32). The HPLC analysis and structure of BBM were shown in Figure 1.

*Toxicity of BBM in zebrafish embryos*

**Figure 2:**
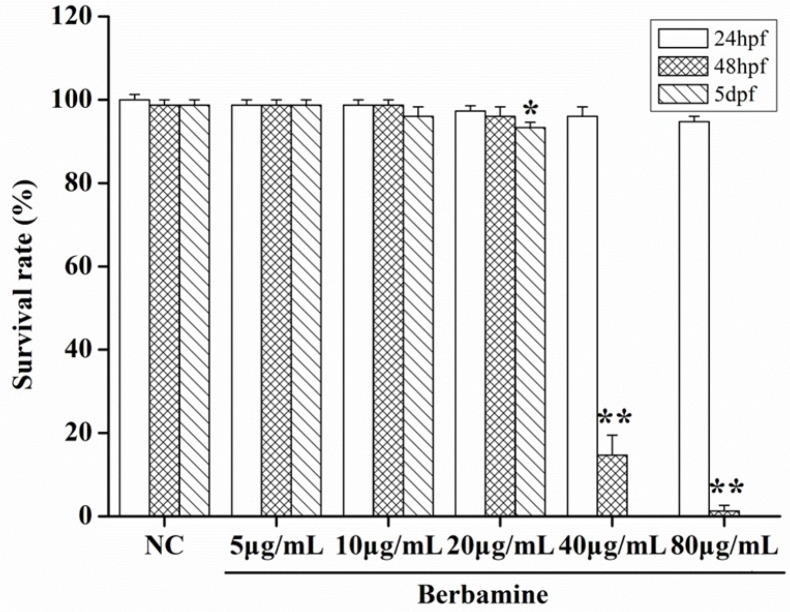
Survival rates after treatment with different concentrations of BBM in zebrafish embryos. NC: normal control; hpf: hours post-fertilization; dpf: days post-fertilization. Data were expressed as means ± SE, n = 50 in each group. * *p*<0.05, **p <0.01 vs. NC group

**Figure 3:**
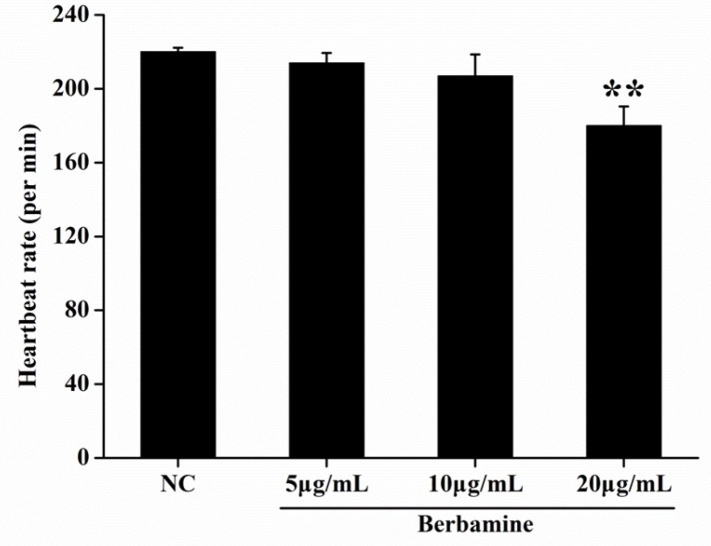
Effect of BBM on heartbeat rate at 5 dpf. The heartbeat was measured at 5 dpf, under the stereomicroscope. The number of atrial and ventricular contraction for 3 min was counted, and the results were represented as the average heartbeat rate (per min). NC: normal control. Data were shown as means ± SE, n = 10 in each group. **p <0.01 vs. NC group

**Figure 4:**
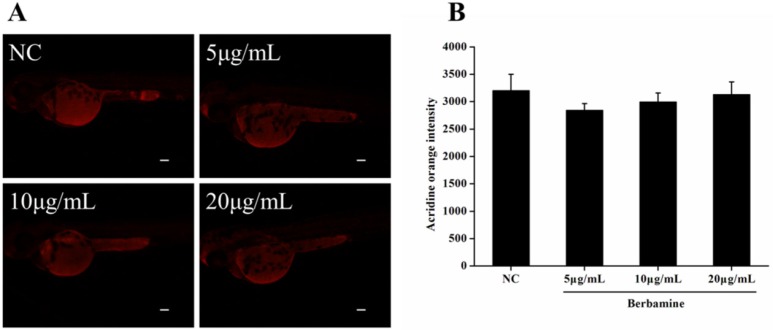
Effect of BBM on cell death in zebrafish embryos. A: fluorescent images after acridine orange staining. B: acridine orange intensity of the larvae. NC: normal control. Data were expressed as means ± SE, n = 10 in each group. Scale bars: 100 μm

In order to determine the toxicity of BBM, we monitored the survival rate and heartbeat rate of zebrafish embryos exposed to BBM with different concentration at a certain time. As shown in Figure 2, in the concentrations range from 5 μg/mL to 80 μg/mL, no death of embryos was observed within 24 hpf. The survival rates of embryos were hardly influenced when treated by BBM with the concentrations less than 20 μg/mL. However, when the dose of BBM exceeded 20 μg/mL, the mortality of zebrafish embryos was increased in a dose- and time-dependent manner (*p *<0.01). At 48 hpf, BBM at a dose of 40 μg/mL and 80 μg/mL showed remarkable adverse effects to zebrafish embryos including developmental malformation and, in some cases, spinal column curving. In the heartbeat test, at 5 dpf, BBM at a dose of 20 μg/mL conspicuously decreased the heartbeat rate of zebrafish embryos as compared with the NC group (*p *<0.01, Figure 3). Acridine orange staining was conducted in the body of the zebrafish (Figure 4A and 4B). The results indicated that there was no significant difference in fluorescence intensity of acridine orange between BBM-treated groups and NC group. These data suggested that less than 20 μg/mL of BBM was relatively safe, which could be used for the further experiments.

*Vascular cholesterol accumulation in larval zebrafish*

**Figure 5:**
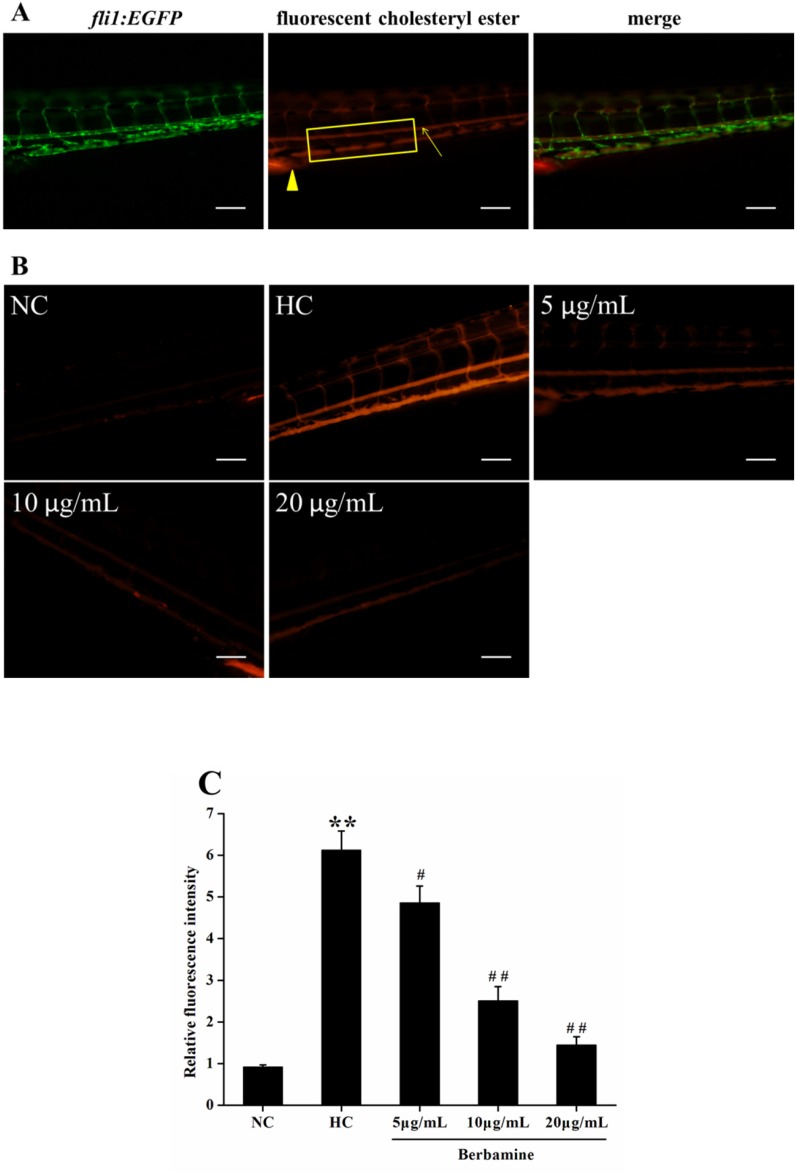
Effect of BBM on vascular cholesterol accumulation in zebrafish larvae. A: fluorescent images of *fli1:EGFP* zebrafish larvae. Arrow: caudal artery; triangle: cloaca. Fluorescence intensity was measured from the cloaca (outlined by rectangle). B: fluorescent images of the caudal artery from tested groups. Lateral views, dorsal is up. C: relative fluorescence intensity of the caudal artery. NC: normal control; HC: high-cholesterol. Values were means ± SE, n = 10 in each group. ^**^p <0.01 vs. NC group; ^#^*p *<0.05, ^##^*p *<0.01 vs. HC group. Scale bars: 100 μm

To explore the effect of BBM on vascular cholesterol accumulation in the caudal artery, live anesthetized zebrafish larvae were imaged using a fluorescence microscope. We also used *fli1:EGFP* zebrafish, which constitutively expressed green fluorescent protein in vascular endothelial cell. Figure 5A showed the fluorescent images of caudal artery (arrow). The caudal artery fluorescence intensity was significantly increased in fish fed with HC diet compared to NC group (*p *<0.01), suggested HC feeding leads to a higher accumulation of cholersterol in zebrafish larvae. However, after treated with BBM for 10 days, BBM dose-dependently reduced the fluorescence intensity in the caudal artery as shown in Figure 5B and 5C. As indicated in the histogram, 5 μg/mL and 10 μg/mL administration of BBM reduced the fluorescence intensity in the caudal artery by 21% (*p *<0.05) and 59% (*p *<0.01), respectively, compared with HC group. Especially, 20 μg/mL treatment of BBM made the fluorescence intensity decreased by 76% as compared to HC group (*p *<0.01). It suggested that BBM could attenuate the accumulation of cholesterol in the blood of zebrafish larvae induced by HC diet.

*Food intake and body weight of adult zebrafish*

**Figure 6:**
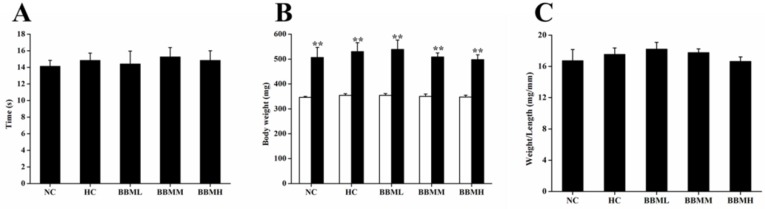
Effects of BBM on food intake and body weight in adult zebrafish. A: the duration of food intake between groups. B: white and black bars showed initial and final body weights, respectively. C: the ratio of body weight to length. NC: normal control; HC: high-cholesterol; BBML: berbamine at low dosage (0.05% w/w); BBMM: berbamine at medium dosage (0.1% w/w); BBMH: berbamine at high dosage (0.2% w/w). Values were means ± SE, n = 20 in each group, ^**^p <0.01 vs. initial weight

During the experimental period, all fish ate up each diet completely. And there was no remarkable difference in food intake (Figure 6A). After 4 weeks feeding, body weight was significantly increased as compared to initial weight in each group (*p *<0.01), no statistical difference among the groups was observed (Figure 6B). The ratio of body weight to length among each group was also not significant (Figure 6C).

*Plasma and liver lipid levels in adult zebrafish*

As shown in Table 2, HC diet led to a significant increase (*p *<0.01) in TC and LDL-c content in plasma compared to the zebrafish fed with control diet. After the administration of BBM, the levels of TC, TG and LDL-c in plasma were declined to different degrees. BBMM decreased the TC and LDL-c levels by 19% (*p *<0.05) and 24% (*p *<0.05), respectively. Among the three therapy groups, BBMH showed the highest lipids lowering activity which reduced the plasma levels of TC, TG and LDL-c by 37% (*p *<0.01), 38% (*p *<0.05) and 28% (*p *<0.01), respectively. However, BBM had no effect on HDL-c level.

**Table 1:** Quantitative real-time PCR primer sequences

**Name**	**Primer Sequences (5’-3’)**	
HMGCR	R: tgcctgcttagtgcatgttc	F: ccagtcaggagtgtccaggt
LDLR	R: gggttgtcaaagtggatgct	F: attcacctgtaccgcctgac
MTP	R: ctctgtgctgccgatctttc	F: tgcagtaacatcagcccaga
CYP7A1a	R: gcagagtgttggcttgtgaa	F: gatcttcccagctctgatcg
GAPDH	R: gccatcaggtcacatacacg	F: gatacacggagcaccaggtt

HMGCR: 3-hydroxy-3-methylglutaryl-coenzyme A reductase;

LDLR: low-density lipoprotein receptor;

MTP: microsomal triglyceride transfer protein;

CYP7A1a: cytochrome P450 polypeptide 1a of subfamily A of family 7;

GAPDH: glyceraldehyde-3-phosphate dehydrogenase.

**Table 2:** Effects of BBM on plasma and liver lipid levels in hypercholesterolemic zebrafish

**Group**	**Plasma lipids profile**				**Liver lipids profile**	
	**TC** **(mmol/L)**	**TG** **(mmol/L)**	**LDL-c** **(mmol/L)**	**HDL-c** **(mmol/L)**	**TC** **(ug/mg** a **)**	**TG** **(ug/mg)**
NC	12.53±3.20	3.44±0.32	3.24±0.37	2.77±0.52	1.91±0.05	1.50±0.13
HC	35.39±0.47**	3.79±0.54	5.70±0.38**	3.61±0.62	3.19±0.10**	2.07±0.20*
BBML	35.06±0.78	3.91±0.33	5.76±0.49	3.88±0.16	3.11±0.11	2.19±0.12
BBMM	28.50±0.90#	3.73±0.12	4.34±0.19#	3.50±0.19	2.82±0.04#	1.94±0.10
BBMH	22.21±2.53##	2.36±0.21#	4.09±0.19##	3.53±0.55	2.36±0.15##	1.57±0.14#

TC: total cholesterol; TG: triglyceride; LDL-c: low-density lipoprotein cholesterol; HDL-c: high-density lipoprotein cholesterol; NC: normal control; HC: high-cholesterol; BBML: berbamine at low dosage (0.05% w/w); BBMM: berbamine at medium dosage (0.1% w/w); BBMH: berbamine at high dosage (0.2% w/w).

Values were means ± SE, n = 20 in each group.

a ug/mg：liver cholesterol or triglyceride/liver weight.

* *p *<0.05,

** *p *<0.01 vs. NC group;

# *p *<0.05,

## *p *<0.01 vs. HC group.

**Figure 7:**
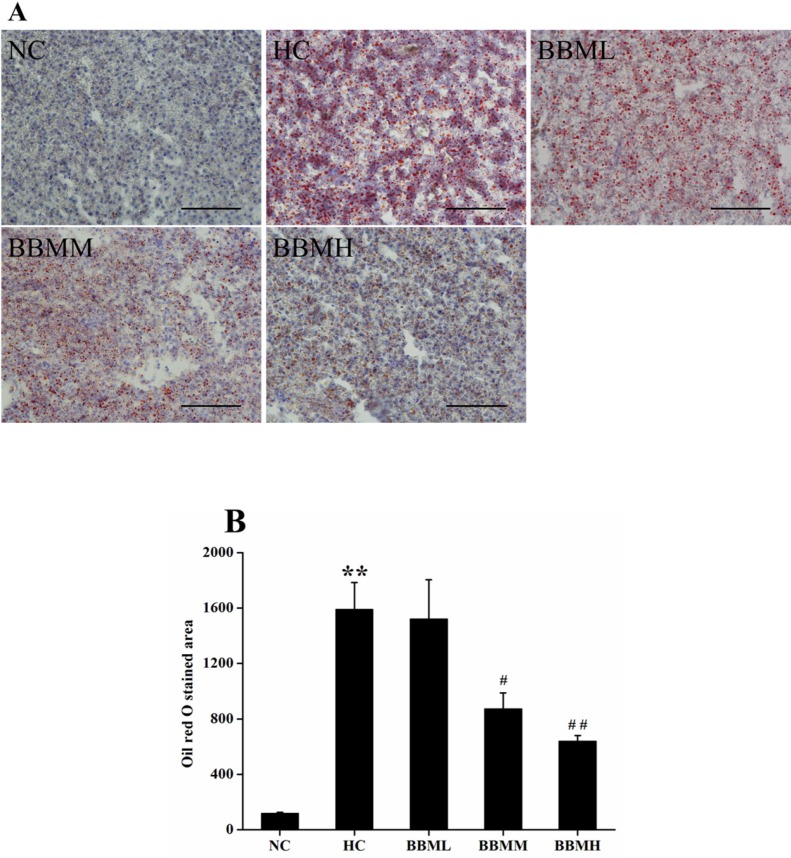
Histological analysis of hepatic tissue in zebrafish. A: images of Oil red O staining in liver. B: Oil red O staind area in the tested groups. NC: normal control; HC: high-cholesterol; BBML: berbamine at low dosage (0.05% w/w); BBMM: berbamine at medium dosage (0.1% w/w); BBMH: berbamine at high dosage (0.2% w/w). Data were shown as means ± SE, n = 10 in each group. ^**^p <0.01 vs. NC group;^ #^*p *<0.05, ^##^*p *<0.01 vs. HC group. Scale bars: 100 μm

**Figure 8:**
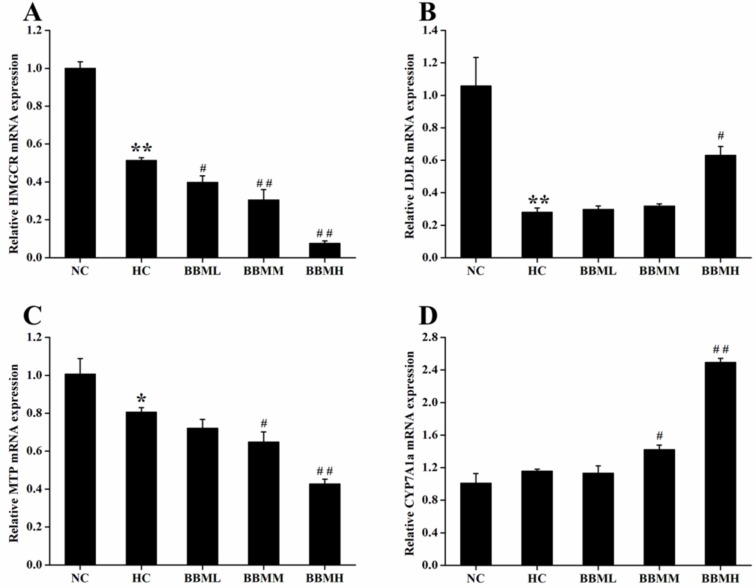
Effects of BBM on expression of genes involved in cholesterol metabolism in liver. A: HMGCR; B: LDLR; C: MTP; D: CYP7A1a. NC: normal control; HC: high-cholesterol; BBML: berbamine at low dosage (0.05% w/w); BBMM: berbamine at medium dosage (0.1% w/w); BBMH: berbamine at high dosage (0.2% w/w). Values were means ± SE, ^*^p <0.05, ^**^p <0.01 vs. NC group;^ #^*p *<0.05, ^##^*p *<0.01 vs. HC group

In liver, the levels of TC and TG in HC group were significantly higher than those in NC group (*p *<0.01 or *p *<0.05). Compared to the HC group, BBM treatment dose-dependently reduced the TC and TG levels in hypercholesterolemic zebrafish. BBMM reduced the TC level in liver by 12% (*p *<0.05) and the high dosage of BBM decreased the TC and TG levels by 26% (*p *<0.01) and 24% (*p *<0.05), respectively. These data indicated the beneficial effects of BBM on plasma and liver lipid profiles in hypercholesterolemic zebrafish.

*Histological analysis*

In an effort to analyze the physiological impact of BBM on the fat accumulation in zebrafish liver, the liver sections were obtained from different groups followed by Oil red O staining (Figure 7A). For quantitative analysis of the lipid content, the Oil red O stained area was measured by Nikon Elements-DR software (Figure 7B). As shown in Figure 7, histological analysis of hepatic tissue indicated that the zebrafish fed with control diet exhibit normal and non-pathological histochemical features, while the fish fed with HC diet demonstrate obviously lipid droplets and abnormal morphology. However, BBM-treated groups showed a reduced Oil red O stained area to a different degree. After 4 weeks treatment, the lipid content in BBMM and BBMH group was decreased by about 45% (*p *<0.05) and 60% (*p *<0.01), respectively, suggested a reduction in hepatic fat storage.

*Expression of mRNA in liver of adult zebrafish*

To determine the potential molecular mechanism by which BBM exerted its anti-hypercholesterolemic effect, the mRNA expression levels of genes associated with cholesterol metabolism in liver were investigated by quantitative real-time PCR.

As shown in Figure 8A, HC diet induced significant decrease in HMGCR mRNA expression (*p *<0.01). In comparison with the HC group, BBM treatment dose-dependently suppressed the HMGCR mRNA expression. All of the three groups inhibited the expression of HMGCR mRNA by 23% (*p*<0.05), 41% (*p *<0.01) and 85% (*p *<0.01), respectively.

LDLR is a cell receptor and involves in the endocytosis of cholesterol. The histogram showed a 0.73-fold decrease (*p *<0.01) of LDLR mRNA in the zebrafish fed with HC diet, possibly due to the high level of cholesterol in the plasma (Figure 8B). However, BBM increased the mRNA level of LDLR, which was monotonously raised along with the increase of BBM dose. And BBMH made LDLR mRNA expression up-regulated by 1.25-fold (*p *<0.05), as compared to the HC group.

MTP encodes an enzyme required for the assembly and secretion of lipoproteins. Results from Figure 8C indicated that the expression of MTP mRNA in HC group was inhibited by 20% (*p *<0.05) compared with the NC group and further reduced in BBM treated groups. Among the tested groups, BBMM and BBMH remarkably down-regulated the MTP mRNA level by 20% (*p *<0.05) and 47% (*p *<0.01), respectively, compared with HC group, which showed a dose-dependent relationship.

CYP7A1a is a key enzyme involves in the conversion of cholesterol to bile acids in liver of zebrafish. After BBM treatment, the expression of CYP7A1a mRNA was increased to different degrees as shown in Figure 8D. As it indicated in the results, BBMM significantly improved the CYP7A1a mRNA expression by 23% (*p *<0.05). Especially, BBMH caused the expression of CYP7A1a mRNA increased by 1.15 times (*p *<0.01) compared with HC group and exhibited a beneficial activity to accelerate the excretion of bile acids.

## Discussion

BBM is one of the minor constituents present in RC (8, 9). Recently, BBM has been demonstrated to possess valuable biological activities including anti-thrombosis, anti-hypertension, anti-arrhythmia, and anti-myocardial ischemia effects (14, 33). However, systematic study of pharmacological mechanism of BBM on hypercholesterolemia is rare. Since zebrafish has similar metabolic patterns to that of human (34, 35), the present study is conducted to evaluate the anti-hypercholesterolemic effect of BBM using zebrafish model. To our knowledge, this is the first attempt to demonstrate the effect of BBM on hypercholesterolemic zebrafish and to provide a rationale for the use of BBM in hyperlipidaemia.

Diet plays an important part in the regulation of cholesterol homeostasis. Consumption of a cholesterol-enriched diet leads to the development of hyperlipidemia, which, in turn, is a major risk factor for atherosclerosis and cardiovascular disease. Recently, a zebrafish model for early atheroschlerosis has been developed by administration of a HC diet (17). Consistent with this report, our study showed that HC diet resulted in a significantly increase of TC and LDL-c levels (182% and 76%, respectively) in zebrafish compared to the NC group. This may because fish favor lipids rather than carbohydrates as the source of energy and are prone to be hyperlipidemic compared to other mammals (36). After the administration of BBM, the elevated levels of TC and LDL-c in hypercholesterolemic zebrafish were tempered to different degrees. BBM at a high dosage down-regulated the TC and LDL-c levels by 37% and 28%, respectively.

The histological analysis of liver sections showed neutral lipid augmented in HC diet fed zebrafish. Whereas reduced Oil red O stained area was observed in BBM treatment groups, suggested a reduction in hepatic fat storage. From this point of view, the results indicated that BBM could alleviate the hepatic steatosis which is caused by increased lipid levels in liver. 

The optically transparent zebrafish embryo and larvae enable *in-vivo* monitoring of vascular processes in transgenic zebrafish or fed with diet supplemented with fluorescent tracers (17). In the caudal artery, the fluorescence intensity of HC diet fed larvae was significantly increased. After concomitant administration of BBM, we observed declined fluorescence intensity in the caudal artery. This demonstrated BBM can reduce the cholesterol accumulation in vascular. By using liquid chromatography-mass spectrometry, Fang et al. reported that zebrafish larvae fed with HC diet for 2 weeks resulted in a 70-fold increase in specific oxidized cholesteryl esters (37). Moreover, Xie *et al*. injected a red fluorescent DiI-LDL into *fli1:EGFP* embryos and found that the concentration of LDL was increased at the vascular wall compared to the center of the lumen, suggested a polarized LDL distribution (38). In human, the majority of serum cholesterol is carried by LDL particles. Therefore, the observed inhibition of fluorescent cholesterol accumulation in the caudal artery with BBM administration may be attributable to the suppression of cholesterol absorption.

Zebrafish expresses all major nuclear receptors, lipid transporters, lipoproteins and enzymes involved in lipid metabolism (34, 37, 39). During the development of zebrafish embryos, the yolk supplies nutrition to the whole body and basically functions as a liver, where all the lipoprotein synthesis occurs. To understand the mechanism behind the decreased lipid levels after BBM administration, we investigated the genes related to cholesterol metabolism and detected the mRNA expression of HMGCR, LDLR, MTP and CYP7A1a in liver. As in mammals, zebrafish MTP functions in the very low-density lipoprotein (VLDL) assembly in the yolk during early development (40). Schlegel *et al*. used Oil red O staining to evaluate the consequence of MTP knockdown on lipid absorption in whole zebrafish larvae. Convincingly, the results manifested that MTP morphants exhibited decreased yolk consumption and incapacity to absorb dietary neutral lipids (41). An initial study executed by OꞌHare *et al*. reported that LDLR deficient zebrafish larvae showed increased LDL-c level and was exacerbated when fed with HC diet. Moreover, the elevated LDL-c was ameliorated when treated with atorvastatin (42). Additionally, ezetimibe, an intestine cholesterol absorption inhibitor, or simvastatin treated zebrafish larvae demonstrated reduced cholesterol level. Moreover, combination of ezetimibe and simvastatin had an additive effect (43).

In this study, hepatic mRNA expression analysis indicated that BBM could affect cholesterol metabolism in multiple ways. We observed that BBM inhibited the HMGCR and MTP mRNA expression in a dose-dependent manner, which could block cholesterol *de novo* synthesis and the secretion of lipoproteins. Moreover, BBM also promoted the CYP7A1a expression to different degrees, which could accelerate the conversion of cholesterol to bile acids. At the same time, compared with the HC group, BBM at a high dosage up-regulated the transcriptional level of LDLR by 125%, which will increase cholesterol transport in liver. Therefore, these findings suggested that the lipid-lowering effect of BBM in HC diet fed zebrafish may be primarily related to the inhibition of cholesterol synthesis and promotion of cholesterol catabolism and thus leading to a decrease in both plasma and liver lipid levels. 

## Conclusion

Present study demonstrated that BBM had a substantial cholesterol-lowering effect on hypercholesteremic zebrafish. BBM treatment improved cholesterol accumulation in the plasma, liver, and vascular system induced by HC diet. The possible mechanism could be attributed to the down-regulation of cholesterol synthesis and assembly or secretion of lipoproteins as well as up-regulation of cholesterol transport and efflux. These findings suggested that BBM was beneficial to hypercholesterolemia.
